# Modeling and Parallel Operation of Exchange-Biased Delta-E Effect Magnetometers for Sensor Arrays

**DOI:** 10.3390/s21227594

**Published:** 2021-11-16

**Authors:** Benjamin Spetzler, Patrick Wiegand, Phillip Durdaut, Michael Höft, Andreas Bahr, Robert Rieger, Franz Faupel

**Affiliations:** Institute of Materials Science, Faculty of Engineering, Kiel University, 24143 Kiel, Germany; pw@tf.uni-kiel.de (P.W.); pd@tf.uni-kiel.de (P.D.); michael.hoeft@tf.uni-kiel.de (M.H.); andreas.bahr@tf.uni-kiel.de (A.B.); rri@tf.uni-kiel.de (R.R.); ff@tf.uni-kiel.de (F.F.)

**Keywords:** magnetometer, delta-E effect, sensor array, magnetoelectric, cantilever, exchange bias

## Abstract

Recently, Delta-E effect magnetic field sensors based on exchange-biased magnetic multilayers have shown the potential of detecting low-frequency and small-amplitude magnetic fields. Their design is compatible with microelectromechanical system technology, potentially small, and therefore, suitable for arrays with a large number N of sensor elements. In this study, we explore the prospects and limitations for improving the detection limit by averaging the output of N sensor elements operated in parallel with a single oscillator and a single amplifier to avoid additional electronics and keep the setup compact. Measurements are performed on a two-element array of exchange-biased sensor elements to validate a signal and noise model. With the model, we estimate requirements and tolerances for sensor elements using larger N. It is found that the intrinsic noise of the sensor elements can be considered uncorrelated, and the signal amplitude is improved if the resonance frequencies differ by less than approximately half the bandwidth of the resonators. Under these conditions, the averaging results in a maximum improvement in the detection limit by a factor of $\sqrt{N}$. A maximum N≈200 exists, which depends on the read-out electronics and the sensor intrinsic noise. Overall, the results indicate that significant improvement in the limit of detection is possible, and a model is presented for optimizing the design of delta-E effect sensor arrays in the future.

## 1. Introduction

The detection of small amplitude magnetic fields is of interest for various fields of application, e.g., in magnetic recording, geomagnetism, and aerospace engineering [1]. Specific engineering and development challenges arise for biomedical applications, such as cell and particle mapping [2,3], magnetomyography [4,5], or magnetocardiography [6,7,8,9]. Such applications are often connected to inverse solution problems that benefit from large arrays with many sensor elements and the possibility of quick spatial field mapping [10,11]. Magnetic flux densities in this field of application are of the order of tens of picotesla and less [12] with frequency components often well below 1 kHz [5,13]. Therefore, research on sensor systems for biomedical applications is devoted to improving the minimum detectable field at low frequencies while minimizing critical parameters such as size, power consumption, and cost [13].

The gold standard for detecting such small magnetic fields is superconducting quantum interference device (SQUID) magnetometry [14,15]. These sensors must be cooled and magnetically well-shielded during operation, which makes them expensive and extensive to operate. Such setups are limited in the number of sensor elements and their minimum distance to the magnetic source. Atomic magnetometers [16,17,18] have been investigated as an affordable alternative to SQUIDs and have achieved limits of detection (LOD) in the $\mathrm{fT}/\sqrt{\mathrm{Hz}}$ regime at low signal frequencies between 1–200 Hz [16]. Despite this progress, atomic magnetometers often require magnetic shielding, and their limited CMOS integrability and downsizing reduce the number and density of sensor elements that can be used in array applications. Miniaturization and MEMS fabrication of atomic magnetometers is currently an active field of research [19,20]. Many magnetometers are being investigated to overcome such limitations [4,15], and an overview and comparison of magnetic field sensors for biomedical applications can be found [13].

In this work, we study magnetic field sensors based on magnetoelectric composite resonators. Previously, sensor systems utilizing the direct magnetoelectric effect were discussed for magnetocardiography [21] and magnetomyography [5], and limits of detection in the low and sub-$\mathrm{pT}/\sqrt{\mathrm{Hz}}$ regime have been reached with a linear response over several orders of magnitude [22]. Magnetoelectric sensors can be produced on a large scale with standard micro-electro-mechanical system (MEMS) technology and dimensions down to the micrometer range [23]. They are potentially cost-efficient, feature low power consumption, and are integrable with CMOS electronics. These aspects make magnetoelectric sensors promising candidates for sensor arrays. On the other hand, detecting small magnetic flux densities is limited to a narrow bandwidth of a few hertz around the resonance frequency, which is usually in the kilohertz regime for millimeter-sized resonators or the megahertz regime for micrometer-sized devices. Such high and narrow frequency regimes are not suitable for many applications [21]. Shifting them down increases the contributions of 1/f noise and requires large resonators with low resonance frequencies, which are susceptible to mechanical vibrations and reduce the spatial resolution.

Delta-E effect magnetometers extend the measurement range of magnetoelectric sensors and shift it to low frequencies while avoiding 1/f noise and keeping the advantages of magnetoelectric composites and the MEMS fabrication technology. In contrast to sensors based on the direct magnetoelectric effect, delta-E effect sensors benefit from high resonance frequencies because they operate on a modulation scheme. The higher resonance frequencies permit miniaturization and render the devices robust against mechanical disturbances. The modulation occurs via the magnetoelastic delta-E effect [24,25,26], i.e., the magnetization induced change of the stiffness of the material, which leads to a detuning of the resonance frequency upon the application of a magnetic field. This detuning can be measured as a change of the electrical admittance of the sensor and causes a modulation of the current through the sensor [27]. Although precursor steps towards the delta-E effect sensor concept were already made in the 1990s [28], it took another two decades until fully integrable delta-E effect sensors [29] were developed based on microelectromechanical magnetoelectric composite cantilevers [26,30,31,32,33,34], plate resonators [35,36], or other designs [37], including macroscopic laminate structures [38,39]. MEMS cantilever sensors achieved LODs < 100 pT/$\sqrt{\mathrm{Hz}}$ in the frequency range from approximately 10–100 Hz [32]. This is currently of a similar order of magnitude as the LODs of some magnetoresistive sensors [40,41]. As an application example of delta-E effect sensors, magnetic particle mapping was recently demonstrated for cell localization [42]. In this setup, the sensor was operated under a magnetic bias field provided by a permanent magnet. Most studies rely on an external magnetic bias field to operate the sensor at an optimum signal-to-noise ratio. Instead of a permanent magnet, the magnetic bias field is often created with external coils. For delta-E effect sensor arrays with many sensor elements, coils and permanent magnets can be inconvenient because their stray fields shift the operation points of adjacent sensor elements, and the additional electrical components increase the volume of the sensor system.

Recently, we demonstrated a first delta-E effect magnetometer based on exchange biased magnetic multilayers that circumvents such complications and still achieves a minimum detection limit of $350\mathrm{pT}/\sqrt{\mathrm{Hz}}$ at 25 Hz [34]. The exchange bias provides an internal bias field and thereby paves the way to flexible and compact delta-E effect sensor arrays with many sensor elements. Only recently were sensor arrays based on magnetoelectric sensor elements reported [43,44,45,46,47], and were limited to direct magnetoelectric detection and were mostly based on macroscopic resonators. A CMOS integrated array of magnetoelastic sensor elements was presented for vector magnetometry [48], but the sensor elements were only characterized individually and without a signal and noise analysis. No attempts of parallel operating delta-E effect sensor elements in array configurations or thorough signal and noise analyses of such have yet been presented.

In this study, we explore the operation of delta-E effect sensor elements in arrays to improve the signal, noise, and limit of detection. Instead of measuring the magnetic field at different locations, spatial resolution can be sacrificed by averaging the outputs of several sensors operating simultaneously. However, the large number of hardware channels required to achieve the desired improvement in the LOD increases the size of the setup and limits the number and density of sensors. As a solution, sensor elements are connected in parallel and operated and read out simultaneously with one set of electronics. This method of parallel operation is accompanied by other complications, and they are analyzed here to identify the potential of such a setup. After presenting the sensor system, which is based on exchange-biased delta-E effect sensors, a signal-and-noise model is developed and validated with measurements. The model is used to analyze the sensor characteristics as functions of the number of sensor elements and variations in the resonance frequency that can occur during fabrication. Implications for the design of delta-E effect sensor arrays are derived and requirements on the reproducibility are identified and discussed.

## 2. Sensor System

**Figure 1:**
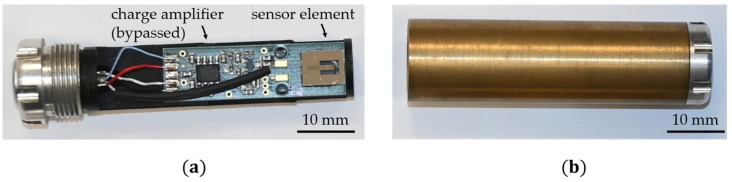
(**a**) Example sensor (without encapsulation) used in this study; it comprises a MEMS-fabricated cantilever resonator as a sensing element mounted on a printed circuit board (PCB). The JFET charge amplifier on the PCB was used in a previous study and is bypassed here and replaced by an external one. (**b**) Brass encapsulation for mechanical protection and electrical shielding during the measurements. Further details are reported in Ref. [34].

In this study, two MEMS fabricated sensor elements are used, based on 50 µm thick poly-Si cantilevers with a length of 3 mm and a width of 1 mm. They are covered with a 4 µm thick exchange-biased magnetic multilayer [49] and a 2 µm thick piezoelectric AlN layer [50] on the top. The AlN layer is contacted via two Ta-Pt electrodes on its top and rear-side for excitation and read-out. The magnetic multilayer is based on alternating antiferromagnetic ${\mathrm{Mn}}_{70}{\mathrm{Ir}}_{30}$ (8 nm) and soft ferromagnetic ${\mathrm{Fe}}_{70.2}{\mathrm{Co}}_{7.8}{\mathrm{Si}}_{12}B_{10}$ (200 nm) layers. In this configuration, the antiferromagnetic layer provides an exchange bias that serves as an internal bias field for the ferromagnetic layer to ensure a nonzero sensitivity without an externally applied magnetic field. Hence, all measurements shown in this study are performed without a magnetic bias field. Details about the layer structure and fabrication process and a comprehensive analysis of sensors with a similar geometry can be found elsewhere [34]. Two sensor elements are mounted on a printed circuit board, respectively, as shown in Figure 1. They are connected in parallel to each other and connected to the input of a low-noise JFET-based charge amplifier [51]. A high-resolution A/D and D/A converter (*Fireface UFX+*, *RME*, Chemnitz, Germany) is used for excitation and read-out (24 bit, 32 kHz). For the measurements, the sensors are placed in a magnetically and electrically shielded setup [52], based on a mu-metal shielding cylinder (*ZG1*, *Aaronia AG*, Strickscheid, Germany), and are located in a copper fleece coated box that is mechanically decoupled to reduce the impact of mechanical vibrations. All magnetic flux densities are applied along the long axis of the cantilever.

## 3. Array Modeling

In an alternating magnetic field, the delta-E effect causes an oscillation of the mechanical stiffness of the cantilever. The response of the cantilever to this stiffness change is damped with increasing magnetic field frequencies because of its mass inertia. Previously, this behavior was modeled with a first-order Bessel filter [27,53], applied to the demodulated simulated output signal of the charge amplifier. Later, a dynamic sensitivity was introduced [54] to consider the low-pass filter characteristics of the sensor as a function of the magnetic field frequency. The dynamic sensitivity was derived from the frequency response of a simple damped harmonic oscillator; however, it is only fully valid if the sensor is excited at its mechanical resonance frequency. For many previously analyzed sensors [33,53,55], this approximation was well justified, as their resonance frequency was close to their optimum excitation frequency, i.e., the excitation frequency with the largest signal-to-noise ratio. This is not a general property of such sensors but depends on their geometry, material, and electrical capacitance. Significant quantitative and qualitative deviations between measurements and simulations can occur if the excitation is not in mechanical resonance [56] (p. 139). In an array, not all sensor elements can be excited in mechanical resonance because of variations in their resonance frequencies that occur during fabrication. In this section, a signal and noise model is developed based on an altered approach, and it permits describing the output signal of an array of *N* sensor elements excited at an arbitrary excitation frequency.

### 3.1. Signal Model

During operation of the sensor array, a sinusoidal voltage $u_{\mathrm{ex}}\left(t\right)$ with amplitude ${\hat{u}}_{\mathrm{ex}}$ and frequency $f_{\mathrm{ex}}$ is applied. It excites the magnetoelectric resonators of each sensor element at, or close to, its respective resonance frequency $f_{r,n}$. In linear approximation, the voltage at the charge amplifier’s output can be described by:

(1) $$u_{\mathrm{co}}\left(t\right)\approx -Z_{f}\left(f_{\mathrm{ex}}\right)\cdot i_{s}\left(t\right).$$

**Figure A1:**
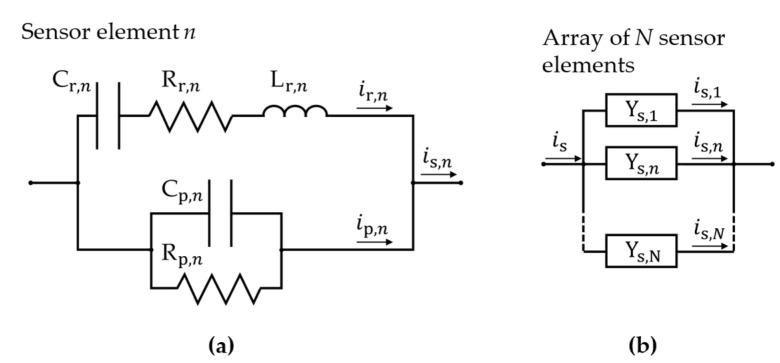
Illustration of (**a**) the modified Butterworth-van Dyke equivalent-circuit model used to describe the sensor elements, with the current $i_{r,n}$ through the resonator LCR-circuit and $i_{p,n}$ as the parallel capacitive pathway, and (**b**) all *N* parallel-connected sensor elements of the array, with the current $i_{s,n}$ through the *n*th sensor element and the total current $i_{s}$ through the array.

In this equation, the time is denoted by *t* and the feedback impedance of the charge amplifier by $Z_{f}$. The current $i_{s}$ through the array of parallel-connected sensor elements can be expressed as the sum of all individual currents $i_{s,n}$ that flow through the respective sensor element *n*. To describe $i_{s,n}$, we use a modified Butterworth-van Dyke (mBvD) equivalent circuit representation, illustrated in detail in Appendix A. It consists of a series resonant circuit with a resistance $R_{r,n},$ inductance $L_{r,n}$, and capacitance $C_{r,n}$ that consider the resonant behavior of the cantilever. The electrodes of each sensor element form a capacitor with the piezoelectric layer. It is described by a capacitance $C_{p,n}$ and resistance $R_{p,n}$, both in parallel to the series LCR-circuit. Further, the current $i_{s,n}$ can be separated into a current $i_{r,n}$, which passes through the resonant LCR circuit, and a current $i_{p,n}$, which passes through the parallel pathway. A sketch of the circuit model is provided in Figure A1 (Appendix A). For *N* parallel-connected sensor elements, $i_{s}$ can be described by:

(2) $$i_{s}\left(t\right)=\overset{N}{\underset{n=1}{\sum }}i_{s,n}=\overset{N}{\underset{n=1}{\sum }}\left(i_{p,n}+i_{r,n}\right)\;.$$

The current $i_{p,n}$ is described as a function of the magnitude $\left|Y_{p,n}\right|$ and the phase angle $\phi_{p,n}≔\mathrm{angle}\left\{Y_{p,n}\right\}$ of the electrical admittance $Y_{p,n}$ of the parallel pathway, and results in:

(3) $$i_{p,n}={\hat{u}}_{\mathrm{ex}}\cdot \left|Y_{p,n}\left(f_{\mathrm{ex}}\right)\right|\cdot \cos\left(2\pi f_{\mathrm{ex}}t+\phi_{p,n}\left(f_{\mathrm{ex}}\right)\right).$$

This current is independent of the magnetic field, and the corresponding electrical admittance $Y_{p,n}\left(f\right)=R_{p,n}^{-1}+2\pi fC_{p,n}$ as a function of frequency *f* is entirely determined by the capacitance $C_{p,n}$ of the respective piezoelectric layer-electrode configuration and its resistance $R_{p,n}$. Similarly, the current $i_{r,n}$ can be described as a function of the magnitude $\left|Y_{r,n}\right|$ and the phase angle $\phi_{r,n}≔\mathrm{angle}\left\{Y_{r,n}\right\}\;$of the magnetic-field and frequency-dependent admittance $Y_{r,n}$ of the resonant circuit of a sensor element *n*. The current $i_{r,n}$ is filtered in the time domain to consider the frequency response of the resonator. We use a second-order digital peaking (resonator) filter with a rational transfer function ${ℋ}_{r}$ that is determined by the resonance frequency $f_{r}$ and the quality factor *Q* (Appendix B). It is given by:

(4) $$i_{r,n}={ℋ}_{r}\left\{{\hat{u}}_{\mathrm{ex}}\cdot \left|Y_{r,n}\left(f_{\mathrm{ex}},B,t\right)\right|\cdot \cos\left(2\pi f_{\mathrm{ex}}t+\phi_{r,n}\left(f_{\mathrm{ex}},B,t\right)\right)\right\}.\;$$

In contrast to $i_{p,n}$, the resonant current $i_{r,n}$ depends on the magnetic flux density $B=B_{0}+B_{\mathrm{ac}}\left(t\right)$, which can be expressed as a static magnetic flux density $B_{0}$, superposed by a small, time *t* dependent contribution $B_{\mathrm{ac}}\left(t\right)$. For small amplitudes ${\hat{B}}_{\mathrm{ac}}$ of $B_{\mathrm{ac}}\left(t\right)$, the admittance $Y_{r,n}\left(f,B\right)$ around $B_{0}$ and at *f* = $f_{\mathrm{ex}}$ can be approximated by a first-order Taylor series:

(5) $$\left|Y_{r,n}\left(f_{\mathrm{ex}},B,t\right)\right|\approx \left|Y_{r,n}\left(f_{r},B_{0}\right)\right|+{\frac{d\left|Y_{r,n}\left(f,B_{0}\right)\right|}{df}|}_{f=f_{\mathrm{ex}}}{\frac{df_{r,n}\left(B\right)}{dB}|}_{B=B_{0}}\cdot B_{\mathrm{ac}}\left(t\right),$$

and

(6) $$\phi_{r,n}\left(f_{\mathrm{ex}},B,t\right)\approx \phi_{r,n}\left(f_{r},B_{0}\right)+{\frac{d\phi_{r,n}\left(f,B_{0}\right)}{df}|}_{f=f_{\mathrm{ex}}}{\frac{df_{r,n}}{dB}|}_{B=B_{0}}\cdot B_{\mathrm{ac}}\left(t\right).$$

Because the damping of the carrier relative to its maximum value at $f_{\mathrm{ex}}=f_{r}$ is already considered by ${ℋ}_{r}$, the zero-order element in the series expansion is taken at $f=f_{r}$ instead of $f=f_{\mathrm{ex}}$. If not stated differently, we always use $B_{0}=0$ because the exchange bias sensors used here do not require an externally applied magnetic bias field.

### 3.2. Definition of Sensitivities

The derivatives in the previous two equations describe the influence of the magnetic field on the electrical admittance and can be referred to as sensitivities. A magnetic sensitivity can be defined as:

(7) $$S_{\mathrm{mag},n}={\frac{df_{r,n}}{dB}|}_{B=B_{0}}\;,$$

and two electrical sensitivities $S_{\mathrm{el},\mathrm{am},n}$ and $S_{\mathrm{el},\mathrm{pm},n}$ as:

(8) $$S_{\mathrm{el},\mathrm{am},n}={\frac{d\left|Y_{r,n}\left(f,B_{0}\right)\right|}{df}|}_{f=f_{\mathrm{ex}}},S_{\mathrm{el},\mathrm{pm},n}={\frac{d\phi_{r,n}\left(f,B_{0}\right)}{df}|}_{f=f_{\mathrm{ex}}}.$$

These definitions of electrical sensitivities differ from previous work [26,53,57], which is further discussed at the end of this section. A normalization, as in Refs. [26,57], is still required to compare the electric and magnetic sensitivity of sensors with different resonance frequencies. After amplification by the charge amplifier, the output signal $u_{\mathrm{co}}\left(t\right)$ is fed into a quadrature amplitude demodulator to obtain the demodulated signal u(t). The amplitude spectrum $\hat{U}\left(f\right)$ of u(t) can then be used to define the voltage sensitivity $S_{V}\left(f\right)$ as a normalized measure for the sensor’s signal response:

(9) $$S_{V}\left(f_{\mathrm{ac}}\right)=\frac{\hat{U}\left(f_{\mathrm{ac}}\right)}{{\hat{B}}_{\mathrm{ac}}}\;\mathrm{with}\;\left[S_{V}\right]=\frac{V}{T}\;.$$

The voltage sensitivity $S_{V}\left(f_{\mathrm{ac}}\right)$ can be estimated by applying a sinusoidal magnetic test signal $B_{\mathrm{ac}}={\hat{B}}_{\mathrm{ac}}\sin\left(2\pi f_{\mathrm{ac}}t\right)$, with well-defined amplitude ${\hat{B}}_{\mathrm{ac}}$ and frequency $f_{\mathrm{ac}},$ to obtain $U\left(f_{\mathrm{ac}}\right)$ from the measurement. With $S_{V}\left(f_{\mathrm{ac}}\right),$ a measure for the smallest detectable magnetic field can be defined. This measure is frequently referred to as limit of detection (LOD) [22,27], equivalent magnetic noise [58,59], or detectivity [40]:

(10) $$\mathrm{LOD}\left(f_{\mathrm{ac}}\right)=\frac{E\left(f_{\mathrm{ac}}\right)}{S_{V}\left(f_{\mathrm{ac}}\right)}\;\mathrm{with}\;\left[\mathrm{LOD}\right]=\frac{T}{\sqrt{\mathrm{Hz}}}\;,$$

where $E\left(f_{\mathrm{ac}}\right)$ is the voltage noise density of u(t) at $f_{\mathrm{ac}}$, after demodulation and measured without any magnetic field applied. The response of Delta-E effect magnetometers to magnetic fields depends on the mutual orientation of the magnetic field and sensor element. Consequently, the sensitivities and LOD (Equations (7)–(10)) are generally functions of the orientation of the magnetic field. Details about the signal-and-noise characterization of ∆E-effect magnetometers can be found elsewhere [27,53,54].

The definitions of the electrical sensitivities (Equation (8)) differ from previous formulations [26,53,57] limited to the special case of one sensor element excited in resonance ($f_{\mathrm{ex}}=f_{r}$). The electrical sensitivities defined within those models use the total sensor admittance $Y_{s}≔Y_{r}+Y_{p}$ instead of $Y_{r}$ to form the derivatives with respect to the frequency. Here, the parallel admittance $Y_{p}$ is considered in the total sensor current $i_{s}$. This definition arises naturally from separating the sensor current into the resonator current and the current though the capacitor, and it is used to consider the response of the resonator to the alternating magnetic field.

### 3.3. Noise Model

**Figure 2:**
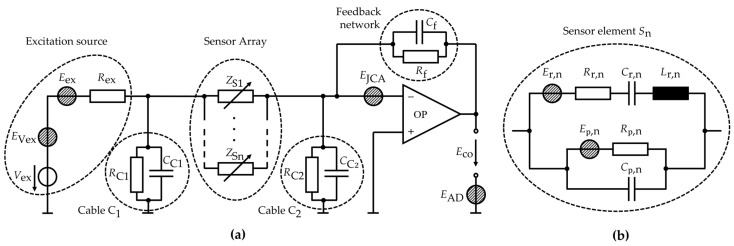
(**a**) Noise model of the sensor system comprising an excitation source, charge amplifier, and *N* sensor elements $S_{n}$ with impedances $Z_{Sn}$, connected in parallel. (**b**) Equivalent circuit noise model of each sensor element $S_{n}$, with resonator intrinsic noise source $E_{r,n}$ and piezoelectric noise source $E_{p,n}$.

In the following, we modify and extend the model presented in Ref. [54] to analyze the noise of the array sensor system and how it is influenced by adding parallel sensor elements. Additional sensor elements are considered and minor noise sources, e.g., of the cables, are omitted. The equivalent circuit noise model is shown in Figure 2 and a summary of the parameters is given in Table A1 in Appendix C. The noise of the excitation source is described by a thermal-electrical noise source $E_{\mathrm{ex}}$ of the D/A converter’s output resistance $R_{\mathrm{ex}}$ and the D/A converter’s quantization noise $E_{\mathrm{Vex}}$. Similarly, $E_{\mathrm{AD}}$ describes the noise that occurs during the analog-digital conversion. The noise source of the JFET charge amplifier is calculated from the model in [51] and is summarized in $E_{\mathrm{JCA}}$. Each sensor element of the array is described by the mBvD equivalent circuit (Figure 2b). For the sensor intrinsic noise of the *n*th sensor element, we consider the thermal-electrical noise source $E_{p,n}$ of the piezoelectric layer and the thermal-mechanical noise source $E_{r,n}$ of the resonator. The value of the thermal-electrical noise sources can be calculated from:

(11) $$E_{x}=\sqrt{4k_{B}TR_{x}}\;\mathrm{with}\;x\;\in \left\{\mathrm{ex},p,r\right\},$$

with Boltzmann’s constant $k_{B}=1.38\times 10^{-23}J/K$ and the temperature T=290 K. The noise source $E_{\mathrm{Vex}}$ and $E_{\mathrm{AD}}$ were obtained from measurements. Here, we consider small excitation amplitudes ${\hat{u}}_{\mathrm{ex}}<100\;\mathrm{mV}$ only and obtain $E_{\mathrm{Vex}}=26.8\;\mathrm{nV}/\sqrt{\mathrm{Hz}}$ and $E_{\mathrm{AD}}=6.9\mathrm{nV}/\sqrt{\mathrm{Hz}}$ in this case. Each noise source is transformed to the output of the charge amplifier to analyze its contribution to the total noise density at the charge amplifier’s output. To simplify the final expressions, the following impedances are defined. The impedance $Z_{s,n}$ of the *n*th sensor is obtained from:

(12) $$Z_{s,n}=Z_{r,n}\|Z_{p,n}\;,$$

(13) $$Z_{r,n}=R_{r,n}+j\omega L_{r,n}+\frac{1}{j\omega C_{r,n}}\;,$$

(14) $$Z_{p,n}=\frac{1}{j\omega C_{p,n}}\|R_{p,n}\;,$$

where || denotes the parallel operator (a||b = (a^−1^ + b^−1^)^−1^).

The total impedance of all *N* sensor elements connected in parallel is:

(15) $$Z_{s}={\left[\overset{N}{\underset{n=1}{\sum }}Z_{s,n}^{-1}\right]}^{-1}\;.$$

The impedance $Z_{C2}$ of the cable with capacitance $C_{C2}$ and resistance $R_{C2}$ between the sensor elements and the charge amplifier is given by:

(16) $$Z_{C2}=\frac{1}{j\omega C_{C2}}\|R_{C2},$$

and the feedback impedance of the charge amplifier by:

(17) $$Z_{f}=\frac{1}{j\omega C_{f}}\|R_{f},$$

with the capacitance $C_{f}$ and the resistance $R_{f}$. The total voltage noise density at the output of the charge amplifier is obtained from the superposition of the individual output referred noise sources,

(18) $$E_{\mathrm{co}}^{2}=E_{\mathrm{co},\mathrm{JCA}}^{2}+E_{\mathrm{co},\mathrm{Vex}}^{2}+E_{\mathrm{co},\mathrm{AD}}^{2}+E_{\mathrm{co},s}^{2}\;,$$

$E_{\mathrm{co},\mathrm{JCA}}$ of the charge amplifier, $E_{\mathrm{co},\mathrm{Vex}}$ of the D/A converter, $E_{\mathrm{co},\mathrm{AD}}$ of the A/D converter, and the contribution $E_{\mathrm{co},s}$ of the parallel sensor elements. These noise contributions are given by:

(19) $$E_{\mathrm{co},\mathrm{JCA}}^{2}=E_{\mathrm{JCA}}^{2}{\left|1+\frac{Z_{f}}{Z_{s}+Z_{C2}}\right|}^{2}\;,$$

(20) $$E_{\mathrm{co},\mathrm{Vex}}^{2}=E_{\mathrm{Vex}}^{2}{\left|\frac{Z_{f}}{Z_{s}}\right|}^{2}\;,$$

(21) $$E_{\mathrm{co},\mathrm{AD}}^{2}=E_{\mathrm{AD}}^{2}\;,$$

(22) $$E_{\mathrm{co},s}^{2}=\overset{N}{\underset{n=1}{\sum }}\left(E_{r,n}^{2}\left|\frac{Z_{f}}{Z_{r,n}}\right|+E_{p,n}^{2}\left|\frac{Z_{f}}{R_{p,n}}\right|\right)\;≔E_{\mathrm{co},r}^{2}+E_{\mathrm{co},p}^{2}.$$

## 4. Characterization and Validation of the Signal-and-Noise Model

In this section, the sensor elements and the array are characterized regarding their impedance, signal, and noise as well as their frequency response. The measurements are compared with simulations to demonstrate the validity of the model. Details on the implementation of the model are given in Appendix D.

### 4.1. Electrical Sensitivity and Admittance Characterization

**Figure 3:**
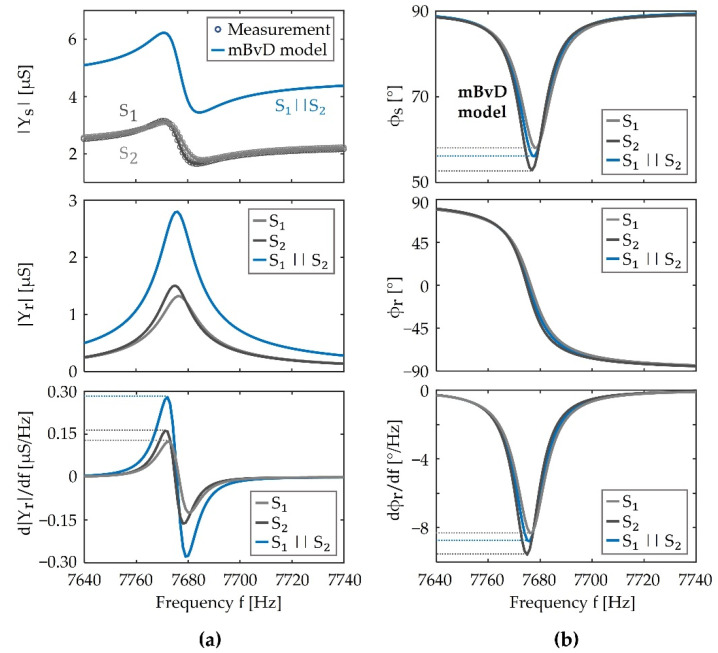
(**a**) Top: magnitudes $\left|Y_{s}\right|$ of the admittance of the sensor elements *S*_1_, *S*_2_ and both connected in parallel (*S*_1_||*S*_2_) measured at an applied magnetic flux density of *B* = 0 and an excitation voltage amplitude of ${\hat{u}}_{\mathrm{ex}}=10\;\mathrm{mV}$, compared with a modified Butterworth-van Dyke (mBvD) equivalent circuit fit; middle: magnitude $\left|Y_{r}\right|$ of the electrical admittance of the LCR series circuit of the mBvD model; bottom: derivative of $\left|Y_{r}\right|$ with respect to the frequency *f*, which we refer to as electrical amplitude sensitivity. (**b**) Top: corresponding phase angles $\phi_{s}$ of the sensor elements obtained from the mBvD model; middle: phase angles $\phi_{r}$ of the admittance of the LCR series circuits; bottom: their derivates, which we refer to as electrical phase sensitivities.

To eventually compare simulations with measurements, the admittance of the sensor elements is characterized. Measurements of the admittance magnitude |$Y_{s}$| as functions of frequency *f* are shown in Figure 3a (top) for the two sensor elements $S_{1}$, $S_{2}$, and both connected in parallel (*S*_1_||*S*_2_). All measurements were made at $B_{0}=0$ and an excitation voltage amplitude of ${\hat{u}}_{\mathrm{ex}}=10\;\mathrm{mV}$. An mBvD equivalent circuit as described earlier and illustrated in Appendix A is fitted to the magnitude data. The parameters obtained from the fit are given in Table A1 in Appendix C. From the mBvD parameters, we obtain resonance frequencies of $f_{r,1}=7674.9\;\mathrm{Hz}$ and $f_{r,2}=7676.5\;\mathrm{Hz}$ and quality factors of $Q_{1}=642$ and $Q_{2}=558$ (equations in Appendix A). This results in resonator bandwidths $f_{\mathrm{BW},n}\approx f_{r,n}/Q_{n}$ of $f_{\mathrm{BW},1}\approx$ 12 Hz $\mathrm{and}\;f_{\mathrm{BW},2}\approx 14\;\mathrm{Hz}$. Hence, the difference in resonance frequencies $∆f_{r}=\left|f_{r,2}-f_{r,1}\right|=1.6\;\mathrm{Hz}$ is significantly smaller than the bandwidth of the sensor elements.

With the mBvD parameters, the phase angle $\phi_{s,n}$ is calculated and plotted in Figure 3b (top). It shows the typical minimum of an electromechanical resonator that is caused by the superposition of the current through the resonator and the current through the parallel capcitance $C_{p,n}$ and resistance $R_{p,n}$. The values of $Y_{s,n}$ and $\phi_{s,n}$ are similar to other electromechanical resonators that have been operated as delta-E effect sensors (e.g., [32,34,54]). Hence, the chosen sensor elements are representative examples. The admittance magnitude $\left|Y_{r,n}\right|$ and phase angle $\phi_{r,n}$ of the series resonance circuit are obtained from the mBvD model by omitting the parallel current $i_{p,n}$ and are plotted in Figure 3a,b (middle). They exhibit the behavior expected from a linear resonator and the main difference between the two sensor elements is the small difference in their resonance frequencies. The electrical sensitivities $S_{\mathrm{el},\mathrm{am},n}$ and $S_{\mathrm{el},\mathrm{pm},n}$ are calculated following the definitions in Equation (8) from the derivaties of $\left|Y_{r,n}\right|$ and $\phi_{r,n}$ with respect to the frequency. They are plotted in Figure 3a,b (bottom). Both sensor elements have similar electrical sensitivities with extrema of $S_{\mathrm{el},\mathrm{am},1}^{\max}\approx S_{\mathrm{el},\mathrm{am},2}^{\max}\approx \pm 0.15\;µS/\mathrm{Hz}$ and $S_{\mathrm{el},\mathrm{pm},1}^{\max}\approx S_{\mathrm{el},\mathrm{pm},2}^{\max}\approx -8.5{}^{\circ}/\mathrm{Hz}$. Note that $S_{\mathrm{el},\mathrm{am},n}=0$ at $f_{\mathrm{ex}}=f_{r,n}$, but $S_{\mathrm{el},\mathrm{pm},n}=S_{\mathrm{el},\mathrm{pm},n}^{\max}$. Because the two sensor elements have very similar resonance frequencies, their total electrical admittance $Y_{s}=Y_{s,1}+Y_{s,2}$ in parallel connection ($S_{1}||S_{2}$) shows qualitatively the same behavior but with a much increased admittance magnitude and electrical amplitude sensitivity by approximately a factor of two compared to the single sensor elements. The corresponding plots are shown in Figure 3. Comparing the magnitude and phase of $Y_{s}$($S_{1}||S_{2}$) emphasizes that an improvement in the sensitivity is only expected for the electrical amplitude sensitivity $S_{\mathrm{el},\mathrm{am}}$, because the magnitudes $\left|Y_{r,n}\right|$ add up. In contrast, the electrical phase sensitivity $S_{\mathrm{el},\mathrm{pm}}$ does not improve, as it results from averaging $S_{\mathrm{el},\mathrm{pm},1}$ and $S_{\mathrm{el},\mathrm{pm},2}$. For a more comprehensive and general discussion, signal and noise must be considered and, in particular, their dependencies on the magnetic field frequency and the differences in resonance frequency. For that, the signal model is validated in the following section.

### 4.2. Frequency Response of the Sensor

**Figure 4:**
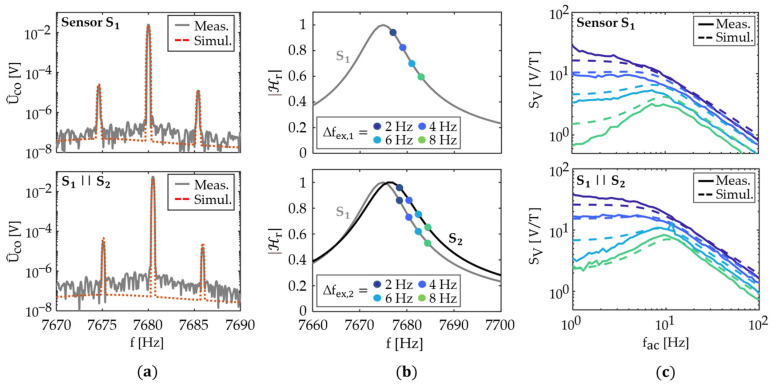
Comparison of simulations with measurements. (**a**) Example amplitude spectrum of the measured and simulated output signal using only the sensor element $S_{1}$ (top) and both sensor elements in parallel $S_{1}||S_{2}$ (bottom) (${\hat{u}}_{\mathrm{ex}}=25\;\mathrm{mV}$). (**b**) Magnitude $\left|{ℋ}_{r}\right|$ of the transfer function ${ℋ}_{r}$ of the resonator used to indicate several excitation frequencies $f_{\mathrm{ex}}$ by $∆f_{\mathrm{ex},1}≔\;f_{\mathrm{ex}}-f_{r,1}$, relative to the resonance frequency $f_{r,1}=7674.9\;\mathrm{Hz}$ of the sensor element $S_{1}$ (top), and $∆f_{\mathrm{ex},2}≔\;f_{\mathrm{ex}}-f_{r,2}$, relative to the resonance frequency $f_{r,2}=7676.5\;\mathrm{Hz}\;$ of the sensor element $S_{2}$ (bottom). (**c**) Measured and simulated voltage sensitivity $S_{V}$ (Equation (9)) as a function of the magnetic field frequency $f_{\mathrm{ac}}$ for the excitation frequencies indicated in (**b**) for the sensor element $S_{1}$ (top) and both sensor elements in parallel $S_{1}||S_{2}$ (bottom) (${\hat{u}}_{\mathrm{ex}}=10\;\mathrm{mV}$).

The electrical sensitivities and sensor parameters found in the previous section are used here in the signal model and the simulations are compared with measurements. In Figure 4a, the spectrum ${\hat{U}}_{\mathrm{co}}$ of the modulated signal is shown from a measurement of the sensor element $S_{1}$ (top) and $S_{1}||S_{2}$ (bottom) using an excitation signal with a voltage amplitude of ${\hat{u}}_{\mathrm{ex}}=25\;\mathrm{mV}$, a frequency $f_{\mathrm{ex}}=7680\;\mathrm{Hz}$ and a sinusoidal magnetic test signal with an arbitrarily chosen frequency of $f_{\mathrm{ac}}=5.8\;\mathrm{Hz},$ and an amplitude of ${\hat{B}}_{\mathrm{ac}}=1\;µT$. Besides the carrier peak at $f_{\mathrm{ex}}$, both spectra show one pair of peaks at $f_{\mathrm{ex}}\pm f_{\mathrm{ac}}$, which corresponds to the modulating signal caused by the magnetic field. Following the magnitude-frequency response of the transfer function of the resonator, the side peak closest to the resonance frequency at $f_{r,1}=7674.9\;\mathrm{Hz}$ ($S_{1}$) is the largest. The signal model fits the measurements very well for magnetic sensitivities of $S_{\mathrm{mag},1}\approx S_{\mathrm{mag},2}=24\;\mathrm{Hz}/\mathrm{mT}$. Considering the normalization required for a comparison [26,57], $S_{\mathrm{mag},n}/f_{r,n}$ is in the typical range expected from similar sensor elements [34,57].

Several $f_{\mathrm{ex}}\neq f_{r}$ are chosen to analyze the sensor’s magnitude-frequency response for operating out of resonance. They are indicated in Figure 4b for $S_{1}$ (top) and $S_{1}$||$S_{2}$ (bottom) as the difference $∆f_{\mathrm{ex},1}≔\;f_{\mathrm{ex}}-f_{r,1}$ of $f_{\mathrm{ex}}$ to the resonance frequency $f_{r,1}$ of $S_{1}$_,_ and the difference $∆f_{\mathrm{ex},2}≔\;f_{\mathrm{ex}}-f_{r,2}$ of $f_{\mathrm{ex}}$ to $f_{r,2}$, respectively. For each excitation frequency, the voltage sensitivity $S_{V}$ as a function of the magnetic field frequency $f_{\mathrm{ac}}$ was measured four times, averaged, and plotted in Figure 4c. As expected, the measurements of both configurations ($S_{1}$ and $S_{1}$||$S_{2}$) show qualitatively the same behavior. For excitation frequencies close to $f_{r}$, the sensor’s voltage sensitivity $S_{V}$ exhibits a low-pass behavior with a maximum voltage sensitivity at the lowest magnetic field frequency $f_{\mathrm{ac}}$.

With an increasing deviation of $f_{\mathrm{ex}}$ from $f_{r}$, the maximum shifts to larger values of $f_{\mathrm{ac}}$ and further reduces its value. The reduction of the voltage sensitivity is caused by a change of the electrical sensitivities as well as the transfer function of the resonator. The model matches the measurements well with deviations mostly smaller than a factor of two and well within the estimated errors of the measurements. In line with the estimation based on the electrical sensitivity in the previous section, the simulations, and measurements of $S_{1}$||$S_{2}$, show an overall improved voltage sensitivity compared to $S_{1}$. A more detailed analysis of this is given in Section 5, where the model is used to estimate the influence of a resonance mismatch for otherwise identical sensor elements.

### 4.3. Validation of the Noise Model

**Figure 5:**
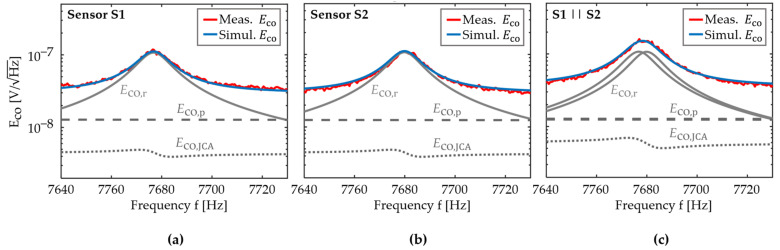
Comparison of the simulated and measured total voltage noise density $E_{\mathrm{co}}$ (Equation (18)) around the sensor’s resonance frequency. The simulated contributions of the thermal-mechanical noise density $E_{\mathrm{co},r}$, the thermal-electrical noise density $E_{\mathrm{co},p}$ of the piezeoelectric layer, and the operational amplifier’s noise density $E_{\mathrm{co},\mathrm{JCA}}$ are shown as well. Measurements and simulations are compared for a sensor system with (**a**) a single sensor element $S_{1}$, (**b**) a single sensor element $S_{2}$, and (**c**) two sensor elements connected in parallel ($S_{1}$||$S_{2}$).

We omit the effect of ${\hat{u}}_{\mathrm{ex}}$ on the quality factor and noise floor for the small ${\hat{u}}_{\mathrm{ex}}$ used in this work, in line with previous investigations [53,54]. Noise measurements are performed for ${\hat{u}}_{\mathrm{ex}}=0$, i.e., the sensor’s input is shortened to ground potential, and data are recorded for 5 min with a sample rate of 32 kHz. The measured noise density spectra are compared with the simulations in Figure 5. The contributions of the sensor intrinsic thermal-mechanical noise $E_{\mathrm{co},r}$, and piezoelectric thermal-electric noise $E_{\mathrm{co},p},$ as well as the operational amplifier’s noise $E_{\mathrm{co},\mathrm{JCA}},$ are shown. The measurements and simulations match well and show what is expected for no excitation, or small excitation amplitudes. Thermal-mechanical noise dominates the noise floor around the resonance frequency and, further away, thermal-electrical noise of the piezoelectric resistance. The maximum noise density peak in Figure 5 is increased by a factor of approximately 1.3 compared to the single sensor elements. This is slightly less than the maximum increase by a factor of $\sqrt{2}$ expected from Equation (22), and it is likely caused by the resonance mismatch.

## 5. Implications for Sensor Arrays

### 5.1. Influence of the Number of Sensor Elements

**Figure 6:**
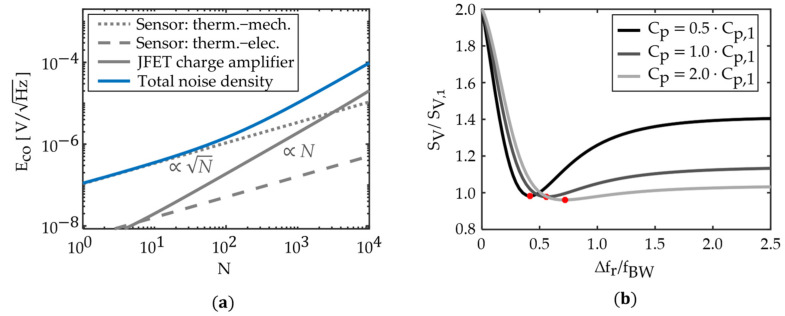
(**a**) Simulation of the voltage noise density at the resonance frequency as a function of the number *N* of the parallel connected sensor elements. Below approximately N=200, the noise level is dominated by the sensor intrinsic thermal-mechanical noise and increases $\propto \sqrt{N}$. At approximately $N>N_{\max}\approx 200$, the noise of the charge amplifier dominates the noise floor and is ∝N. No significant improvement in the signal-to-noise ratio is expected for $N>N_{\max}\approx 200$ because the signal amplitude increases ∝ *N* as well. (**b**) Simulated gain $S_{V}/S_{V,1}$ in the voltage sensitivity $S_{V}$ of a system with two parallel connected sensor elements relative to the voltage sensitivity $S_{V,1}$ of a single sensor element as a function of the difference $∆f_{r}$ in their resonance frequencies, normalized to the bandwidth $f_{\mathrm{BW}}≔f_{r}/Q$ of the resonator. Examples are shown for three different parallel capacities, expressed as multiples of the parallel capacitance $C_{p,1}$ of the sensor element $S_{1}$. The minima are indicated with red dots.

The noise model is used to estimate the influence of the number *N* of sensor elements on the minimum detectable magnetic flux density. First, we consider the ideal case of identical sensor elements described with the mBvD parameters of the sensor $S_{1}$. In this case, the signal magnitude increases linearly with *N*. The change of the total voltage noise density is less trivial because the various noise contributions depend differently on *N*. A simulation of the voltage noise density at the resonance frequency is shown in Figure 6 as a function of *N*. While the sensor intrinsic thermal-mechanical noise and thermal-electrical noise increase $\propto \sqrt{N}$, the noise of the JFET charge amplifier is ∝N. This linear relationship can be explained with the expression for the noise gain $\left|1+Z_{f}/\left(Z_{s}+Z_{C2}\right)\right|$ of the amplifier in Equation (19). According to this expression, the noise gain is in good approximation ($Z_{s}\gg Z_{C2},1)$ inversely proportional to the impedance $Z_{s}$ of the array. Each additional sensor element increases the capacitance and reduces $Z_{s}$ (Equation (15)), and therefore, the noise gain increases linearly with *N* if all sensor elements are identical. The thermal-mechanical noise and the thermal-electrical noise of the amplifier dominate the noise floor at different *N* owing to their different dependencies on *N.* At small *N,* the thermal-mechanical noise dominates the noise floor and the LOD $\propto 1/\sqrt{N}$ can be improved by adding sensor elements. At large *N*, the noise contribution of the amplifier dominates and no improvement in the LOD can be achieved because signal and noise are both ∝N. A transition region exists at intermediate values of *N* where the improvement in LOD decreases continuously with *N*. This transition region is approximately around $N=200≔N_{\max}$ for the set of sensor parameters considered.

### 5.2. Influence of Resonance Frequency Mismatch

The reproducibility of sensor elements can be considerably impaired by the relaxation of small stress during fabrication [60] and by small variations in the resonator geometry. The condition $f_{\mathrm{ex}}=f_{r}$ cannot be fulfilled simultaneously for all *N* sensor elements because both mechanisms cause a distribution in resonance frequency $f_{r}$. At this point, it remains unclear to what extent such a distribution impairs signal, noise, and LOD. However, knowing the tolerable variation in resonance frequency is important for the design of sensor arrays because it imposes limitations on the resonator geometry and on the tolerances of the fabrication process.

First, the voltage sensitivity $S_{V}$ is calculated as a function of the resonance frequency mismatch because it must be known to estimate the LOD (Equation (10)). The model sensor system considered comprises two sensor elements connected in parallel. Both sensor elements have identical resonance frequencies and sensitivities, and they are described by the same set of mBvD equivalent circuit parameters of the sensor element $S_{1}$. The resonance frequency $f_{r}\propto 1/\sqrt{L_{r}C_{r}}$ of one sensor element is altered by increasing the mBvD parameters $L_{r}$ and $C_{r}$ in equal ratios. This keeps the quality factor $Q\propto L_{r}/C_{r}$ constant (Appendix A), and it causes only a negligible change in the bandwidth for the range of resonance frequencies tested. For each difference $∆f_{r}$ in the two resonance frequencies, we simulate the output signal using a magnetic test signal with a frequency of $f_{\mathrm{ac}}=1\;\mathrm{Hz}$, and calculate the voltage sensitivity $S_{V}$ (Equation (9)). This procedure is repeated for three different example capacities $C_{p}$ with values that are multiples of the parallel capacitance $C_{p,1}$ of the sensor element $S_{1}$. We define the sensitivity gain$\;S_{V}/S_{V,1}$ by the voltage sensitivity $S_{V}$ of the two parallel sensor elements, normalized to the voltage sensitivity $S_{V,1}$ of the single sensor element. The results are plotted in Figure 6b as functions of $∆f_{r}$ normalized to the bandwidth $f_{\mathrm{BW}}=f_{r}/Q$ of $S_{1}$.

For all three values of $C_{p}$, the sensitivity gain reaches a maximum value of $S_{V}/S_{V,1}=2$, when the resonance frequencies are identical $∆f_{r}/f_{\mathrm{BW}}=0$. It decreases to a minimum of around $S_{V}/S_{V,1}=1$ at roughly $∆f_{r}/f_{\mathrm{BW}}=0.5$, indicated in Figure 6b with red dots. For larger $∆f_{r}/f_{\mathrm{BW}}$, $S_{V}/S_{V,1}$ increases slightly but it does not reach its maximum value again. The influence of the parallel capacitance $C_{p}$ on $S_{V}/S_{V,1}$ is distinct but it does not change the curves qualitatively in the considered range. A larger $C_{p}$ reduces $S_{V}/S_{V,1}$ at high $∆f_{r}/f_{\mathrm{BW}}$ and shifts the location of the minimum to a larger $∆f_{r}/f_{\mathrm{BW}}$; hence, it slightly broadens the peak around the maximum of $S_{V}/S_{V,1}$. Consequently, the condition of approximately $∆f_{r}/f_{\mathrm{BW}}<0.5$ (depending on $C_{p})$ should be fulfilled to increase the signal magnitude in an array with two sensor elements. This condition can be expressed as:

(23) $$∆f_{r,\mathrm{BW}}≔\frac{∆f_{r}}{f_{\mathrm{BW}}}\approx \frac{∆f_{r}}{f_{r}}\;\cdot Q<0.5\;.$$

The simulations in Figure 6b demonstrate that Equation (23) is not a strict criterium because the locations of the minima on the $∆f_{r,\mathrm{BW}}$-axis vary by up to 50% for different tested values of $C_{p}$ (e.g., for $C_{p}=2C_{p,1}$ the minimum is at $∆f_{r,\mathrm{BW}}\approx 0.75$). The exact location of the minimum depends on the contribution of the current through the LCR pathway to the total sensor current, relative to the capacitive contribution of $C_{p}$. For all practical purposes, these two contributions can hardly be varied independently because changing the parallel capacitance is accompanied by a change of the excitation efficiency, e.g., by altering the electrode geometry [32] or the piezoelectric material [33].

Not only for the voltage sensitivity, but also the sensor intrinsic thermal-mechanical noise referred to as the output follows the transfer function of the resonator; this is demonstrated with the measurements and simulations in Figure 5. Therefore, the LOD is constant if the sensor intrinsic thermal-mechanical noise dominates the noise floor, which is typically fulfilled for excitation frequencies $f_{\mathrm{ex}}$ within the bandwidth of the resonator and sufficiently small magnetic field frequencies $f_{\mathrm{ac}}$. This conclusion is in line with other experimental results [55] (Figure 6) and does still hold for two parallel operating sensor elements with different resonance frequencies. Consequently, it is also LOD$\left(∆f_{r,\mathrm{BW}}\right)=const.$ if the thermal-mechanical noise dominates the voltage noise density at $f_{\mathrm{ex}}+f_{\mathrm{ac}}$. The frequency band around the resonance frequency where the LOD is constant depends on the difference between the thermal-electric noise level and the resonance-amplified thermal-mechanical noise level and changes with *N*. For the sensors analyzed, this range is approximately $<f_{\mathrm{BW}}\;$around $f_{r},$ as shown in Figure 5.

## 6. Discussion and Conclusions

The signal-and-noise model developed matches well with measurements on exchange-biased sensor elements operated separately and in parallel in a setup with a single oscillator and amplifier. The model does still hold for excitation frequencies out of resonance and more than one sensor element. Hence, two major limitations of previous models have been solved and a tool is presented that can further support the design of delta-E effect sensors and sensor arrays. From the good match of the model and consistency with noise measurements, we find that the sensor intrinsic noise in our setup can be considered as uncorrelated, despite the parallel connection of sensor elements and their operation and read-out by a single oscillator and single amplifier. This is an essential precondition for improving the sensor performance by operating in parallel while using fewer electronic elements to keep the setup compact. Additional requirements were identified, which must be fulfilled to improve the signal and the limit of detection by operating many sensor elements in parallel. For the given sensor system, no significant improvement in the limit of detection can be achieved if a maximum number $N_{\max}\approx 200$ of sensor elements is exceeded. Above this number, the noise contribution of the amplifier dominates the noise floor and increases, like the signal amplitude ∝N. Below, the sensor intrinsic noise dominates around the resonance and increases merely $\propto \sqrt{N}$, which results in $LOD\propto 1/\sqrt{N}$. With the given $N_{\max}$, this would correspond to an LOD improvement by a factor of approximately 14. The value of $N_{\max}$ depends on the contribution of the thermal-mechanical noise relative to the thermal-mechanical noise. Therefore, $N_{\max}$ can potentially be improved by reducing the noise contribution of the charge amplifier. The proportionalities found do only hold strictly if all sensor elements are identical. Simulations of the voltage sensitivity confirmed that the improvement in signal amplitude depends significantly on the difference in the resonance frequencies of the sensor elements. It vanishes at a bandwidth normalized resonance frequency difference of approximately $∆f_{r,\mathrm{BW}}\approx 0.5,$ depending on the value of the parallel capacitance of the sensor element. Consequently, a large signal improvement by parallel operation requires tight tolerances on the resonance frequency and, therefore, on the reproducibility provided by the fabrication process. Because the sensor intrinsic noise follows the same resonator transfer function as the signal, we expect the LOD to be constant with $∆f_{r,\mathrm{BW}}$ for sufficiently small $∆f_{r,\mathrm{BW}}$, and here at approximately $∆f_{r,\mathrm{BW}}<2$. This value depends on the level of the thermal-mechanical noise, relative to the thermal-electrical noise of the piezoelectric layer and the noise contribution of the amplifier.

In conclusion, a model was presented that overcomes previous limitations and can be used to explore the signal and noise characteristics of delta-E effect sensor arrays. The results from measurements and simulations indicate that large arrays of parallel operating sensor elements can be an option to improve the signal and limit of detection in the future.

## Data Availability

The data presented in this study are available on reasonable request from the corresponding author.

## Appendix A. Equivalent Circuit Model

The equivalent circuit model used to describe the electrical admittance of each sensor element is illustrated in Figure A1a and the structure of the modeled sensor array in Figure A1b.

The following equations are used to estimate the resonance frequency $f_{r,n}$ and the quality factor $Q_{n}$ of the *n*th sensor element:

(A1) $$Q_{n}=\frac{1}{R_{r,n}}\sqrt{\frac{L_{r,n}}{C_{r,n}}}\;,$$

and

(A2) $$f_{r,n}=\frac{1}{2\pi \sqrt{L_{r,n}C_{r,n}}}\;.$$

## Appendix B. Transfer Function of the Resonator

The frequency response of the resonator is modeled as second-order infinite impulse response (IIR) peaking filter with the transfer function ${ℋ}_{r}\left(z\right)$ [61]:

(A3) $${ℋ}_{r}\left(z\right)=\frac{n_{1}+n_{2}z^{-1}+n_{3}z^{-2}}{d_{1}+d_{2}z^{-1}+d_{3}z^{-2}}\;,$$

and the components $n_{i}$ of the numerator coefficients as well as $d_{i}$ of the denominator coefficients, which are functions of the quality factor *Q* and the resonance frequency $f_{r}$ [62]:

(A4) $$n=\left(\begin{array}{c} 1-g\\ 0\\ g-1 \end{array}\right)\;,\;\;d=\left(\begin{array}{c} 1-g\\ -2g\cdot \cos\left(\pi f_{r}\right)\\ 2g-1 \end{array}\right)\;.\;$$

To ensure a gain of −3 dB at the bandwidth, the factor *g* is set to:

(A5) $$\;g={\left[1+\sqrt{2}\cdot \tan\left(\frac{\pi }{2}\frac{f_{r}}{Q}\right)\right]}^{-1}\;\;.$$

## Appendix C. System Parameters

In the following Table A1, the model parameters of the sensor system and the equivalent circuit parameters of the two sensor elements *S*_1_ and *S*_2_ are summarized.

**Table A1:** Parameters of the equivalent circuit noise model and the modified Butterworth-van Dyke (mBvD) model.

Component	Parameter	Value	Parameter	Value
Excitation	$R_{\mathrm{ex}}$	75 Ω		
$\mathrm{Cable}\;C_{1}$	$R_{C1}$	147 MΩ	$C_{C1}$	208 pF
$\mathrm{Cable}\;C_{2}$	$R_{C2}$	184 MΩ	$C_{C2}$	36 pF
$\mathrm{Sensor}\;\mathrm{element}\;S_{1}$	$R_{p,1}$	295 MΩ	$C_{\mathrm{ME},1}$	47.157 pF
	$R_{r,1}$	663.47 kΩ	$C_{r,1}$	48.725 fF
	$L_{r,1}$	8.826 kH	$f_{r,1}$	7674.9 Hz
$\mathrm{Sensor}\;\mathrm{element}\;S_{2}$	$R_{p,2}$	310 MΩ	$C_{\mathrm{ME},2}$	48.568 pF
	$R_{r,2}$	755.96 kΩ	$C_{r,2}$	49.112 fF
	$L_{r,2}$	8.753 kH	$f_{r,2}$	7676.4 Hz
Amplifier	$R_{f}$	5 GΩ	$C_{f}$	30 pF

## Appendix D. Implementation of the Model

The equations, which describe the signal-and-noise model (Equations (1)–(22)), are implemented in MATLAB (The MathWorks, Inc., Natick, MA, USA). The voltage at the charge amplifier’s output $u_{\mathrm{co}}\left(t\right)$ is calculated in the time domain using Equations (1)–(6), and the electric sensitivities (Equations (7) and (8)) obtained from the impedance measurements in Section 4.1. Estimated magnetic sensitivities of $S_{\mathrm{mag},1}\approx S_{\mathrm{mag},2}=24\;\mathrm{Hz}/\mathrm{mT}$ are used, which is in the typical range expected from similar sensor elements [34,57]. The simulated time domain signal is demodulated with a quadrature amplitude demodulator and subsequently converted to the frequency domain using Welch’s method [63]. From the power spectral density estimate, we calculate the amplitude spectrum $\hat{U}\left(f\right)$ of the demodulated signal u(t) and the voltage sensitivity following the definition provided by Equation (9). For the noise simulations, Equations (11)–(22) are implemented to obtain the voltage noise density at the output of the charge amplifier in the frequency domain.
